# An unexpected cause of gastric submucosal lesion

**DOI:** 10.1590/S1679-45082017AI3772

**Published:** 2017

**Authors:** Rachid Guimarães Nagem

**Affiliations:** 1 Universidade Federal de Minas Gerais, Belo Horizonte, MG, Brazil.

**Figure 1:**
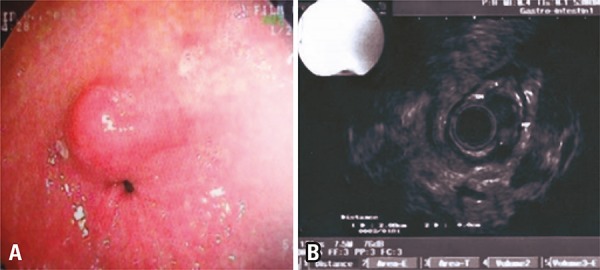
(A) Endoscopy showing a bulge in gastric antrum adjacent to the pylorus. (B) Endoscopic ultrasound revealed the lesion as compatible with a gastrointestinal stromal tumor, but abdominal computed tomography was recommended

**Figure 2:**
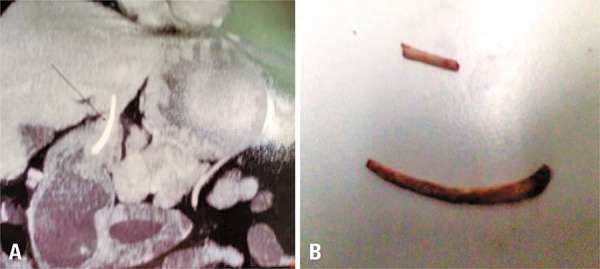
(A) Abdominal computed tomography showing the lesion involving the stomach and liver. (B) The ingested foreign body: a chicken bone

A 52-year-old asymptomatic patient underwent a follow-up endoscopy for Barrett’s esophagus. His exam showed a 1.5cm bulge in the gastric antrum (Figure 1A). The patient was referred for endoscopic ultrasound, which considered the lesion as a gastrointestinal stromal tumor (Figure 1B), but not a typical one, a computed tomography (CT) of the abdomen was, then, suggested. The CT revealed a heterogeneous lesion involving the gastric antrum and the left lateral segment of the liver (Figure 2A). The exploratory laparotomy revealed the lesion to be a chicken bone (Figure 2B). The postoperative was uneventful except for a suppurative infection on the surgical site.

Gastric submucosal lesions are normally mesenchymal in the origin and include gastrointestinal stromal, leiomyomas, leiomyosarcomas, neuroendocrine neoplasms and schwannomas.^1^ Endoscopic ultrasound is currently considered the standard approach for evaluating intramural gastric lesions.^2^ Gastrointestinal stromal can originate in any part of the gastrointestinal tract. In gastrointestinal stromal (60% of all gastrointestinal stromal tumor), surgical resection is normally recommended. Small tumors (<2cm) with no signs of malignancy (ulceration, bleeding, irregular margin, necrosis and cystic change) can be managed with active surveillance. However, there is potential for malignancy in any gastrointestinal stromal tumor, regardless of size.^1,3-5^

Perforation of the digestive tract caused by ingested foreign bodies, on the other hand, is rare. Most of these foreign bodies pass through the digestive tract, and less than 1% of them cause perforation.^6^ For unknown reasons, some of them perforate the gastric wall and become lodged at the left lobe of the liver.^7^Removal can be achieved by endoscopy, laparoscopy or laparotomy. It is important to mention that ingestion of foreign bodies often occurs in people who use dental prosthesis, as occurred with our patient. Prostheses hamper oral sensibility. And, they may, not only, be swallowed themselves but also facilitate the act of swallowing some other foreign body.^7,8^
